# Channelizing the red blood cell: molecular biology competes with patch-clamp

**DOI:** 10.3389/fmolb.2015.00046

**Published:** 2015-08-11

**Authors:** Lars Kaestner

**Affiliations:** Research Center for Molecular Imaging and Screening, Medical School, Saarland UniversityHomburg, Germany

**Keywords:** erythrocyte, Gardos channel, Piezo1, patch-clamp techniques, ion channels

Both patch-clamp and molecular biology provide powerful tools to investigate ion channels. However, the approaches couldn't be more different. While patch-clamp probes protein function on the scale of a single cell or even down to a single molecule, molecular biology includes techniques that identify the protein, and requires mostly cell populations. According to current knowledge and compared to other cell types, red blood cells (RBCs) possess a rather small variety of ion channels. Nevertheless, both techniques are still revealing new channels. The challenge is to keep the results of both methods in agreement. Such consistency can be shown for a number of channels; just to name successful examples, the voltage-dependent anion channel (VDAC) (Bouyer et al., 2011, 2012) and the NMDA-receptor (Makhro et al., 2013; Hänggi et al., 2014). However, providing a general correlation between patch-clamp and molecular biology derived results has had limited success (Kaestner, 2011; Bouyer et al., 2012). One reason for this difficulty is that techniques themselves or their particular application have certain shortcomings.

Patch-clamp, although an extremely powerful technique to investigate RBCs (Hamill, 1983), is in the RBC research field sometimes presented in a rather fragmentary way. An example is a channel, published in 2000, that was supposed to be induced in RBCs by the malaria parasite (Figure 2 in Desai et al., 2000). In contrast, a channel with very similar properties had already been described in healthy RBCs in 1989 (Figure 6 in Schwarz et al., 1989). After years of debate (for examples, see Egée et al., 2002; Huber et al., 2002; Staines et al., 2003, 2007), it became accepted that the increased conductance in malaria infected RBCs was mediated by endogenous ion channels of RBCs (Bouyer et al., 2007).

For molecular biology-based investigations, RBCs impose a twofold challenge:

- Most genetic approaches are limited to precursor cells, because mammalian RBCs contain neither a nucleus nor ribosomes as a translational machinery.
- It appears to be extremely difficult to isolate pure preparations of RBCs (Minetti et al., 2013). Even in cell preparations filtered on cellulose (Beutler et al., 1976), RNA of tyrosine phosphatase (CD45—a marker for non-RBCs) was found in next generation sequencing. Only further fluorescence-activated RBC sorting revealed CD45-free preparations.

Therefore, proofs for the molecular identity of ion channels in RBCs often include indirect methods.

For example, the Gardos channel, named after an effect initially described by George Gardos based on flux-experiments (Gardos, 1958), was the first channel shown in human RBCs by patch-clamp using single channel recordings (Hamill, 1981). The Gardos channel was later identified to be encoded by the KCNN4 gene (K_Ca_3.1protein, also called hSK4 channel) (Hoffman et al., 2003). Although the RT-PCR of reticulocytes and the Western blots of RBCs look convincing one needs to consider point (ii) above. All other arguments such as Northern blots of human erythroid progenitor cells or properties of heterologously expressed KCNN4 vs. KCNN3 (Hoffman et al., 2003) are indirect in nature. On the patch-clamp side one can find numerous single channel recordings of the Gardos channel (Hamill, 1981, 1983; Grygorczyk et al., 1984; Schwarz et al., 1989), but whole cell recordings are almost missing. Some electrophysiologists even believe the Gardos channel is unmeasurable in the whole-cell configuration of RBCs. I am only aware of one paper in which the authors were courageous enough to publish whole-cell recordings of the Gardos channel in human RBCs (Kucherenko et al., 2013). The lack of more attempts is not surprising considering the estimation of the number of channels per cell based on single channel recordings at approximately 10 (Grygorczyk et al., 1984), which renders whole-cell recordings difficult.

Another example is Piezo1—this mechano-sensitive channel was only recently discovered (Coste et al., 2010) and associated with the anemic disease hereditary xerocytosis (HX) due to mutations of Piezo1 found in HX patients (Zarychanski et al., 2012). Pharmacological modulations in patch-clamp experiments suggest that Piezo1 may also contribute to P_sickle_ in sickle cell disease RBCs (Bae et al., 2011; Ma et al., 2012; Gallagher, 2013). Beside all these findings and a biophysical characterisation of Piezo1 in heterologous expression systems (Bae et al., 2011; Gottlieb and Sachs, 2012), the channels' direct functional or molecular proof in human RBCs remains rather elusive—patch-clamp recordings in HX RBCs lack (statistical) comparison in healthy controls (Figure 2 in Archer et al., 2014). Although Piezo1 abundance and function in mouse RBCs has been shown (Cahalan et al., 2015), so far I have not seen any convincing Piezo1 protein data, such as immunocytochemistry or Western blots, based on human RBCs. However, indirect evidence, e.g., measurements of Gardos channel activity induced by membrane deformations (Dyrda et al., 2010), where deformations are likely to activate Piezo1 eventually triggering Gardos channel activity, contribute to the overall picture.

The intention to present these prominent examples is not to doubt the existence of the Gardos channel or the Piezo1 in RBCs, but to illustrate the difficulties in revealing channel identities or saying it with other words: bringing electrophysiology and molecular biology into agreement. To achieve this goal it seems compulsory to consider some points when investigating ion channels in RBCs:

- Functional studies are always most convincing. Beside patch-clamp recordings those include fluorescence-based methods and tracer flux experiments. Conviction increases if it can be proved that effects originate exclusively from RBCs [see point (ii) above]. This condition is relatively easily met when experiments are performed on single cells under visual inspection, such as patch-clamp or fluorescence imaging.
- Cell population measurements require purification efforts. Centrifugation based methods are insufficient (Minetti et al., 2013). Additional filtering, e.g. through cellulose (Beutler et al., 1976), improves the situation. Filtering should be followed by a gelatine zymography (Achilli et al., 2011), which works fine for human RBCs but is insufficient for mouse RBCs. Quantification through assays detecting tyrosine phosphatase is even better. For really pure RBC preparations high quality sorting procedures based on CD45 antibodies should be implemented.
- Patch-clamp recordings require a full characterisation based on ion selectivity and other biophysical or pharmacological parameters. Channel identification should not preferentially rely on the appearance of traces or the general compatibility of the trace with the hypothesis (e.g, Kaestner and Bernhardt, 2002; Locovei et al., 2006; Archer et al., 2014).

Although sometimes one gets the impression that when RBCs are concerned, molecular biology competes with patch-clamp as indicated by the title, it is obvious and necessary that both techniques need to synergistically complement each other to effectively reveal the complexity of RBCs.

## Conflict of interest statement

The author declares that the research was conducted in the absence of any commercial or financial relationships that could be construed as a potential conflict of interest.
